# Lumbar Spinal Stenosis: Pathophysiology, Biomechanics, and Innovations in Diagnosis and Management

**DOI:** 10.26502/fjsrs0082

**Published:** 2025-02-18

**Authors:** Alexander Abdou, Samuel Kades, Tariq Masri-zada, Syed Asim, Mo’men Bany-Mohammed, Devendra K. Agrawal

**Affiliations:** 1 Department of Translational Research, College of Osteopathic Medicine of the Pacific, Western University of Health Sciences, Pomona, California 91766 USA.

**Keywords:** Conservative Treatment, Degenerative Changes, Interspinous Devices, Laminectomy, Ligamentum Flavum Hypertrophy, Laminoplasty, Lumbar Spinal Stenosis, Lumbar spinous process-splitting laminoplasty, Minimally Invasive Surgery, Neurogenic Claudication, Spinal Biomechanics, Stem Cell Therapy

## Abstract

Lumbar spinal stenosis (LSS) is a common condition caused by the narrowing of the spinal canal, resulting in compression of neural and vascular structures. This compression leads to symptoms such as claudication, paresthesia, and lower extremity weakness. LSS is the leading cause of low back pain and functional limitations, affecting over 103 million people worldwide. Degenerative changes, including ligamentum flavum hypertrophy, facet joint osteoarthritis, and intervertebral disc degeneration, are the primary contributors to LSS. Additional factors, such as genetic predisposition, congenital abnormalities, and autoimmune conditions, are also emerging as contributors. A major challenge in managing LSS lies in differentiating it from other causes of neurogenic symptoms and low back pain while devising an appropriate treatment plan from the wide array of conservative and surgical options available. Minimally invasive surgical techniques, such as lumbar spinous process-splitting laminoplasty and partial facetectomy, are often compared to the gold standard laminectomy with or without fusion. Surgical interventions offer significant improvements in pain relief, disability, and quality of life within 3–6 months; however, these benefits often diminish after 2–4 years. Contrasting evidence demonstrates that long-term outcomes of non-surgical treatments, such as physical therapy, pharmacological management, and lifestyle modifications, are often comparable to surgical modalities. Emerging therapies, including interspinous devices and stem cell therapy, show promise but require further research. Managing LSS requires a multidisciplinary approach tailored to patient-specific factors, including age, comorbidities, and functional goals. Future research should aim to improve diagnostic accuracy, refine surgical techniques, and explore innovative therapies to enhance outcomes for patients with LSS.

## Introduction

Lumbar stenosis is caused by the narrowing of the spinal canal, leading to impingement of neurological and vascular structures and precipitating low back pain. The narrowing of the spine can be at the cervical, thoracic, or lumbar levels, and the presentation differs in correspondence to the neurologic functions controlled at each level. In lumbar stenosis, the leading type of spinal stenosis, the pain often radiates down one or both hips and legs and is also associated with paresthesia and weakness of the lower extremities, often relieved by forward flexion or sitting. Other neurologic deficits and complications include fecal or urinary incontinence and in extreme cases, lower extremity paralysis. The classic symptoms of lumbar spinal stenosis (LSS) are precipitated by prolonged activity and daily biomechanical movements that favor spinal extension.

The narrowing of the spinal canal in LSS does not only compress neural structures but can also impact vascular structures, leading to restricted blood flow and venous congestion. This vascular compromise contributes to ischemia of the nerve roots, which may manifest as neurogenic claudication—a hallmark symptom of LSS, particularly aggravated by physical activity [1]. The reduced blood supply compromises neural function and amplifies pain and fatigue, especially during activities that increase metabolic demand in the nerves, such as walking or standing.

The prevalence of lumbar spinal stenosis is difficult to establish due to the lack of a universally accepted definition for the condition, but it is estimated that symptomatic cases worldwide are at 103 million people [2]. LSS affects more than 200,000 people in the United States and is thought to be among the leading causes of spinal surgery in patients above the age of 65 as of 2016 [3,4]. The Framingham study, a cross-sectional observational study done on LSS, revealed that 4.7% of patients had relative (10–12 cm spinal canal narrowing) congenital LSS and 2.6% of patients had absolute (less than 10 cm spinal canal narrowing) congenital LSS. In comparison, 22.5% of patients had relative acquired LSS and 7.3% of patients had absolute acquired LSS. Acquired LSS was most prevalent in patients aged 60 to 69, with relative and absolute LSS being 47.2% and 19.4% respectively [5].

Lumbar spinal stenosis is most commonly caused by age-related osteoarthritic changes such as spondylosis with varying severities depending on the dimensionality and degree of impingement. A recent systematic review of ten studies on the relationship between degenerative processes found a prevalence of up to 54% comorbidity of LSS with knee or hip ostearthritis, at a median age of 66 years [6]. In addition to coexisting OA, risk factors for degenerative LSS include age, increased body mass index (BMI), greater vertebral body size, and smaller anterior-posterior bony canal diameters [7,8]. In patients with relatively varying arthritic changes and varying degrees of impingement of spinal nerves, it becomes difficult to clinically diagnose LSS as the condition is not easily visualized in radiographs, and commonly presents symptomatically.

Although less common, autoimmune conditions, which include rheumatoid arthritis and ankylosing spondylitis are possible etiologies that lead to LSS. Rheumatoid arthritis more commonly leads to cervical spinal stenosis, however in a study of 107 subjects, it was found that 57% presented with lumbar radiologic findings and symptoms [9]. Ankylosing spondylitis, often discovered early in adulthood, is another autoimmune condition with a predilection for damage to the lumbosacral spine and surrounding soft tissues, associated with LSS [10]. Non-arthritic secondary causes of LSS include tumors of the spine and fractures of the spine in the setting of trauma. Ossification defects of the surrounding spinal ligaments and bony complications such as Paget’s disease of the bone can also precipitate LSS in later adulthood. Even hormonal changes associated with exogenous and endogenous steroid production can also lead to the formation of epidural lipomatosis, or fatty deposits in the bone, which leads to LSS [1], further demonstrating the complexity of the etiology of this disease.

Congenital LSS presents at a younger age and involves multiple levels with few degenerative changes. It can be caused by a shorter pedicular length which causes a smaller cross-sectional spinal canal area, predisposing these patients to earlier complaints of neurogenic claudication [11]. Other inherited conditions, such as scoliosis and achondroplasia, can lead to the narrowing of the spinal canal and earlier presentation of LSS. There are a vast set of treatment options available for LSS which are highly dependent on the symptoms as well as the severity and degree of impingement on neurological structures. The most common treatments include lifestyle modification, pharmaceutical aids, physical therapy, spinal bracing, transcutaneous electrical nerve stimulation, neuromodulation, epidural steroid injections, and surgical interventions [3].

The purpose of this review is to dive deeper into the key aspects of lumbar stenosis including the pathophysiology, biomechanical changes, diagnostic methods, and various treatment modalities. We also compared surgical and nonsurgical outcomes of spinal stenosis in young vs older patients (above and below 50 years of age) to help guide future medical providers in clinical decision-making in the best interest of patients.

### Pathophysiology

Lumbar spinal stenosis comprises central canal, lateral recess, and foraminal subtypes, each characterized by unique pathophysiological mechanisms attributed to the region-specific narrowing of the spinal canal. Central canal stenosis refers to the narrowing of the central spinal canal, typically involving the cauda equina in the lumbar region. The primary pathophysiological mechanisms are associated with degenerative changes, such as hypertrophy of the ligamentum flavum, disc bulging, and osteoarthritic alterations in the facet joints [12]. Ligamentum flavum hypertrophy occurs due to collagen remodeling and increased mechanical load over time, resulting in thickening that reduces the central canal space [13]. Additionally, disc degeneration and vertebral instability can lead to degenerative spondylolisthesis, wherein a vertebra slips forward, further narrowing the canal and increasing the likelihood of nerve compression [14]. These changes primarily lead to symptoms such as neurogenic claudication, which presents as lower extremity pain, numbness, or weakness relieved by lumbar flexion. Lateral recess stenosis is defined by narrowing of the lateral portions of the spinal canal, through which nerve roots pass before exiting the vertebral foramen. Narrowing of the lateral portions is often due to age-related degenerative changes, including facet joint hypertrophy, intervertebral disc degeneration, and osteophyte formation, collectively reducing the lateral recess space.

The development of acquired LSS is a multifactorial process and understanding the pathophysiology is crucial to developing a plan for patients. Degenerative, a form of acquired lumbar stenosis, is a progressive disease that involves all aspects of the spine, presenting with different symptomatology depending on the region of the spine affected. The relative instability of the spine begins with degeneration of the intervertebral disc, causing hypermobility of the vertebral segments. Hypermobility of the vertebral segments leads to increased pressure on the posterior facet joints, leading to a narrower space between the discs, an altered angle of extension, and an enlargement of the facet joints, especially the superior articular process. Over time, these joints can become stiff and even fuse, a process called ankylosis [15].

Additional mechanisms also contribute to the pathophysiology of acquired lumbar spinal stenosis. For example, ligamentum flavum hypertrophy and joint tropism are two mechanisms described in the literature that are directly correlated to the development of LSS. Aleksic et al. [16] demonstrated in their study of 60 patients, evenly divided between patients with lumbar discus hernia and lumbar spinal stenosis, ligamentum flavum hypertrophy was prevalent in all 30 patients with LSS demonstrating the direct association between the two conditions. Additionally, histological analysis revealed increased collagen content and decreased elastic fibers in the ligamentum flavum of LSS patients, leading to reduced elasticity and increased stiffness. These degenerative changes in the ligamentum flavum are significant contributors to the pathophysiology of LSS, emphasizing the role of ligamentum flavum hypertrophy in the development of the condition [16]. In a recent study, they demonstrated a series of genetic factors that predisposed patients to the various pathological processes, which include vertebral body and facet joint osteophyte proliferation, ligamentum flavum hypertrophy, and intervertebral disk degeneration that were directly contributory to the pathogenesis of LSS [17]. They discovered a series of 295 genes associated with the pathological disruptions affecting skeletal muscle and 79 genes associated with whole blood that predisposed patients to LSS [17].

Facet joint tropism refers to the asymmetry in the orientation of the facet joints on either side of the spine. This asymmetry can lead to uneven distribution of mechanical loads, potentially contributing to degenerative changes in the lumbar spine. Research has explored the relationship between facet joint tropism and LSS. For example, a study titled “Facet joint tropism, pelvic incidence and facet joint osteoarthritis in lumbar spinal stenosis” investigated this association. The researchers found that facet joint tropism is significantly associated with the development of facet joint osteoarthritis, which plays a crucial role in the pathogenesis of LSS. The study suggests that the asymmetrical orientation of facet joints may lead to uneven mechanical stress, accelerating degenerative changes and contributing to spinal canal narrowing [18].

In another study examined association between facet tropism and vertebral rotation in patients with degenerative lumbar disease and corroborated these findings, demonstrating that facet tropism is directly associated with vertebral orientation, further contributing to spinal instability by altering the natural biomechanics of the spine, compounding stenosis and contributing to symptom severity [19].

**Figure 1:**
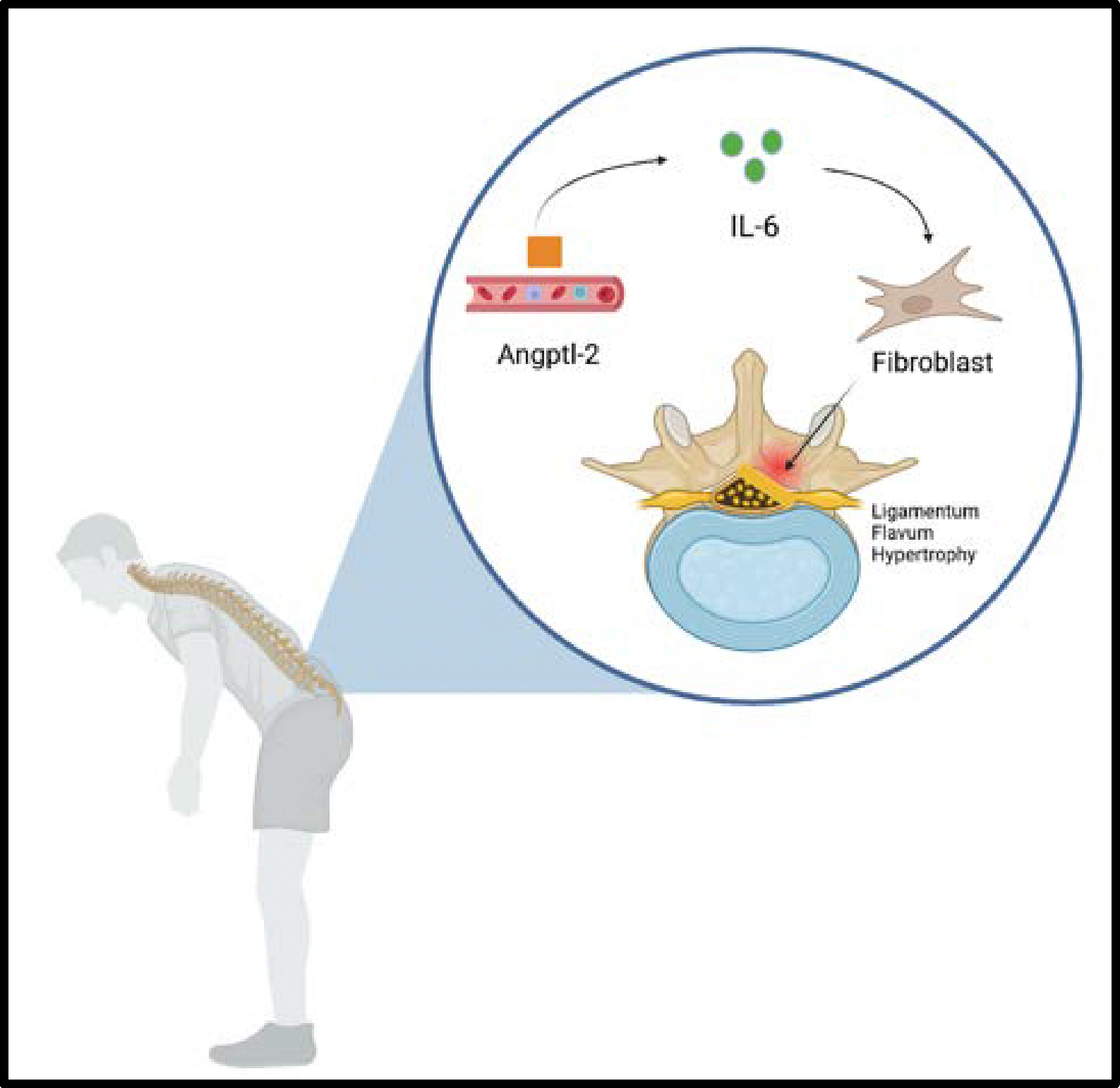
Angiopoietin-like Protein 2 (Angptl-2) induces IL-6-mediated fibroblast activation, leading to ligamentum flavum hypertrophy and spinal canal narrowing, contributing to neurogenic claudication and postural adaptation.

The literature demonstrates that chronic inflammation and fibrosis also play a significant role in the pathophysiology of lumbar spinal stenosis. Several histological studies have revealed increased pro-inflammatory markers and matrix remodeling enzymes in the ligamentum flavum and facet joints, leading to tissue thickening and reduced spinal flexibility. In another investigation examining the effect of angiopoietin-like protein 2 (Angptl2) and the role of interleukin-6 (IL-6) on the inflammatory conditions in the ligamentum flavum in the pathogenesis of lumbar spinal canal stenosis, it was demonstrated how Angptl2 impacts the pathophysiology of LSS (Figure 1). Angptl2, which is a member of the angiopoietin-like protein family, is known to act as an inflammatory mediator. The researchers found that Angptl2 levels are significantly elevated in hypertrophied ligamentum flavum tissue compared to non-hypertrophied tissue in patients with LSS [20]. This elevated Angptl2 expression appears to directly contribute to inflammation by upregulating the expression of IL-6, a cytokine associated with chronic inflammatory responses.

Interleukin-6, a pro-inflammatory cytokine, is a major factor in promoting fibrosis and tissue thickening. When Angptl2 activates IL-6 expression in the ligamentum flavum, it initiates a cascade of cellular responses that increase collagen deposition and fibroblast activity. These processes lead to excessive fibrosis, which results in the loss of elasticity and thickening of the ligamentum flavum. This thickened ligament then encroaches into the spinal canal space, contributing to the neural compression characteristic of LSS [21].

Lumbar spinal stenosis has been linked to congenital etiologies that have led to earlier onset of symptoms. The disease is common, with one study of 191 subjects revealing that absolute lumbar spinal stenosis was seen in 2.6% of patients with congenital implications. Absolute lumbar spinal stenosis had a 7.3% incidence rate in the acquired group [5]. In a comprehensive review discussing developmental lumbar stenosis, it was revealed that patients who develop this tend to have shorter bony canal diameters compared to control subjects [22]. Pedigree analysis reveals that developmental lumbar stenosis follows an autosomal dominant inheritance pattern. There were also significantly shorter pedicle lengths when compared to normal subjects [23,24]. Regarding symptoms and presentation, they present similarly to patients with acquired lumbar spinal stenosis. However, patients will present with an accelerated onset when compared to unaffected subjects.

Studies reveal that mutations in transforming growth factor-beta, and bone morphogenetic protein-2 are also implicated in congenital lumbar spinal stenosis. When mutated, these genes will alter the ossification of the posterior longitudinal ligament. Another gene that is implicated in lumbar spinal stenosis is the fibroblast growth factor receptor 3 (FGFR3), a gene heavily implicated in bone elongation. A few other genes that are implicated include collagen 1A1 (COL1A1) and collagen 1A2 (COL1A2), which lead to intervertebral disc herniation thus leading to lumbar spinal stenosis [25].

### Clinical Presentation in different age groups

Acquired Lumbar Spinal Stenosis typically presents in the sixth or seventh decade of life [26]. It commonly presents with back pain, sciatica, and weakness. A study analyzing 68 patients found that 93% complained of pain, 63% complained of numbness and 43% complained of weakness. Symptoms are usually bilateral and more frequently affect the entire leg as opposed to a single nerve root, which was only seen in 6% of cases [27]. Claudication is also very commonly seen with LSS, as one systematic review found that claudication was present in 82% of patients [28]. Patients with claudication typically report relief with flexion and increased pain with extension. Relief with sitting is also a key differentiating factor between LSS and nonspecific low back pain, as the latter typically worsens with prolonged sitting. Examination of the low back will often show reduced mobility, limited extension, and hamstring tightness. Neurologic examination is typically normal. While many patients with LSS are only symptomatic while active, more severe nerve root involvement can lead to neurologic deficits. These patients may show focal weakness, decreased reflexes, and paresthesia in one or more spinal roots. The study above found that deep tendon reflexes were absent at the ankle in 43 percent of patients, and at the knee in 18 percent of patients [27].

Congenital Lumbar Spinal Stenosis occurs at an earlier age, normally in the 4th or 5th decade. This stenosis is caused by shorter pedicular length as opposed to progressive degeneration. Patients with congenital stenosis will often have multilevel involvement and fewer degenerative changes. Clinically, congenital and acquired lumbar spinal stenosis present similarly. (11)

### Diagnosis of Lumbar Stenosis

The diagnosis of LSS is a multifactorial process that includes patient history, physical examination, imaging modalities, and other diagnostic tests. There is no universally established guideline for diagnosing LSS, however, the North American Spine Association established radiographic imaging as the key noninvasive test in diagnosing LSS [29]. The presence of neurogenic symptoms has been known to help physicians diagnose patients with manifestations suspicious of LSS. Posture has a relationship with the presence of neurogenic claudication symptoms due to the mechanical compression of the spinal nerves in the canal. Extension of the back has been known to exacerbate back pain while flexion has been known to alleviate it in patients with LSS [30].

Several symptoms could arise from LSS, and in a recent study, the most common symptoms that clinicians used to diagnose this condition included low back pain in 58.6% of patients, neurogenic claudication in 43.7%, and paresthesia in the lower limbs 35.6% [31]. Neurogenic claudication is the presence of intermittent leg pain, aches, or shocks while walking due to impingement of spinal nerves. Many patients complain of increased pain in the lower back when walking which is also relieved by flexing forward or sitting down also known as the shopping cart sign across literature.

Muscle wasting of the extensor digitorum brevis (EDB) is a significant indication in those who have LSS. The EDB is a muscle on the dorsal surface of the foot that originates on the lateral surface of the foot innervated by the L5/S1 nerve root. This muscle extends the first four metatarsophalangeal joints and has been postulated to have statistical significance in helping to diagnose LSS. EDB wasting with a diagnosis of LSS was found in 60% of patients unilaterally and 30% bilaterally [32].

LSS has manifestations like cauda equina syndrome (CES) leading to urinary or bowel dysfunction, saddle anesthesia, and lower extremity weakness. CES is a more severe condition that requires urgent surgical intervention to prevent permanent incontinence or neurological dysfunction. CES is most associated with a lumbar disc bulge manifesting as severe back pain or sciatica along with one of the three: saddle anesthesia, bowel or bladder dysfunction, or sexual dysfunction with a neurologic deficit in the lower limbs [33]. The symptoms of these two diseases have a lot of overlap so thorough and prompt diagnosis with imaging, magnetic resonance imaging (MRI) being the gold standard, is necessary to differentiate the two. The presence of waxing and waning and slow onset of these neurological symptoms is also more suggestive of an LSS pathology over CES [34].

The physical exam is an imperative component in determining the presence of LSS in a patient. The straight leg raise test also known as the Lasegue sign is a nonspecific exam used by many clinicians to assess radiculopathy within the lumbar spinal region. The exam is performed by having the patient lay supine, with the affected limb, and the knee extended and raised; the exam is then performed again with the knee flexed. A positive straight leg raise is the reproduction of radicular pain within the affected leg. The location of the radicular pain is associated with the affected spinal root. L4 reproduces pain down the buttock, lateral, and medial thigh. L5 reproduces pain radiating down the buttock, posterior thigh, and lateral calf [35]. Although it could be useful in the assessment of LSS, the straight leg raise test is non-specific.

The Romberg test is a commonly used assessment for detecting LSS. During this test, patients stand still with their eyes closed while the examiner evaluates their balance. A positive result is indicated by unsteadiness or compensatory movements to maintain balance. The Romberg test has a reported specificity of 91% for LSS. Additionally, a wide-based gait is a highly specific physical exam finding, with a specificity of 97%. When evaluating a patient with lower back pain and neurogenic claudication, a thorough physical exam before imaging is essential to accurately assess and rule out other potential causes of the patient’s symptoms [1].

### Biomechanical Changes in Patients with Lumbar Spinal Stenosis

The biomechanics of LSS involve complex interactions between spinal loading, pelvic tilt, body weight, gait, posture, muscle activity, cerebrospinal fluid dynamics, and surgical interventions. Loading of the spine, particularly during walking, can exacerbate symptoms in LSS patients. Mousavi et al. [36] found that lumbar spine loading during symptomatic walking increased by an average of 7% compared to asymptomatic walking, suggesting that spine loading plays a significant role in symptom provocation. This increased loading can lead to greater compressive forces on the spinal structures, contributing to pain and functional limitations. Penning highlighted that axial loading and retroflexion naturally narrow the spinal canal, which can exacerbate symptoms in patients with stenosis [37].

**Figure 2:**
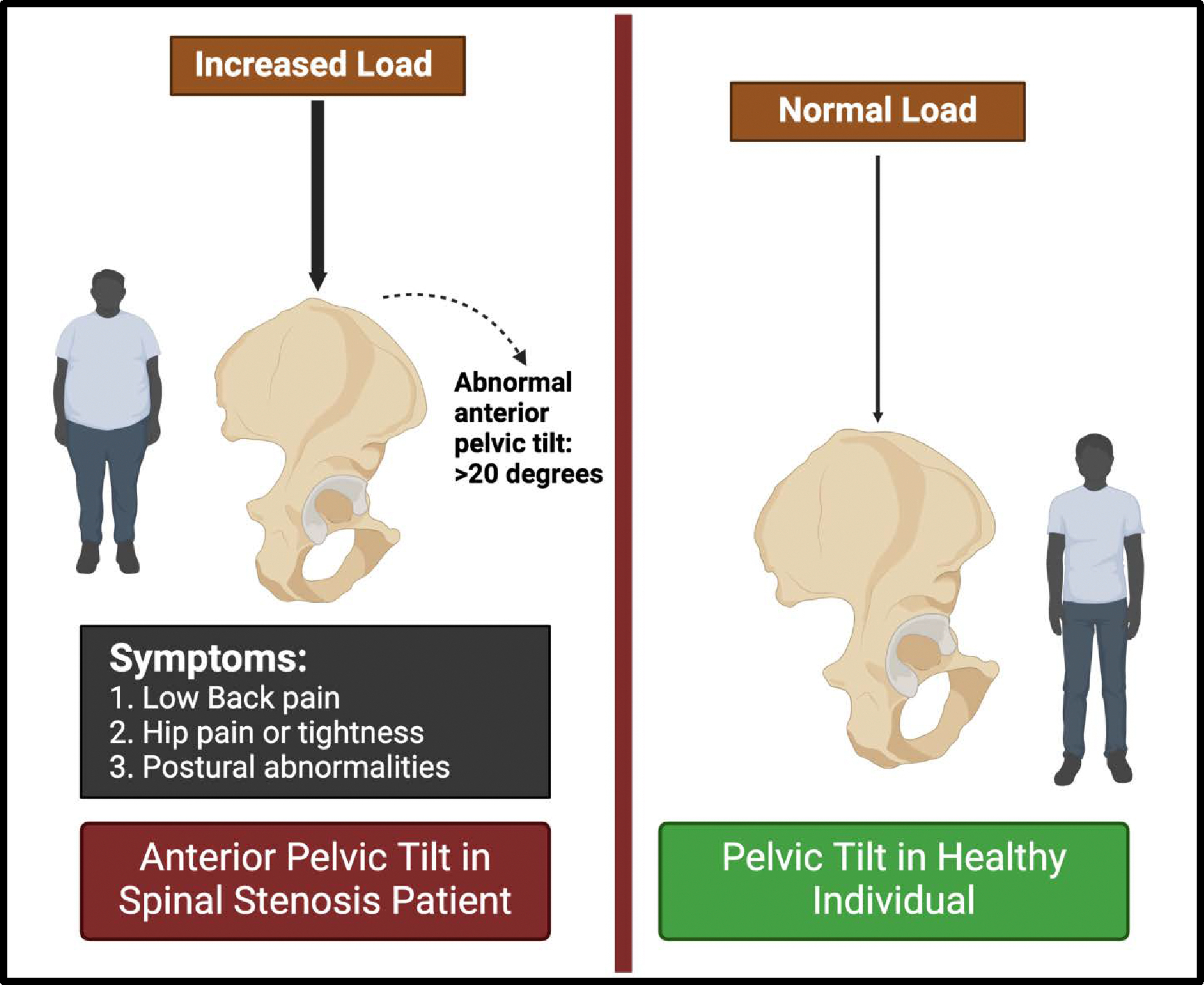
Pelvic tilt comparison: Abnormal anterior tilt (>20°) in lumbar spinal stenosis causes pain and posture issues, unlike proper alignment in healthy individuals.

Pelvic tilt, both anterior and posterior, can affect the biomechanics of the lumbar spine. Kuwahara et al. [38] demonstrated that an increase in anterior pelvic tilt during gait loading was positively correlated with the aggravation of low back pain in LSS patients. Conversely, a smaller anterior lumbar tilt was associated with less aggravation of low back pain, indicating that pelvic alignment can influence symptom severity (Figure 2).

Gait and posture adaptations are common in LSS patients. Perring et al. demonstrated significant alterations in clinically measurable gait parameters in patients with LSS compared with healthy subjects, highlighting the impact of LSS on gait [39]. Igawa et al. [40] identified that patients with LSS might adopt a trunk-flexed posture during walking to alleviate symptoms by increasing the spinal canal diameter. However, not all patients use this strategy, and some may maintain an upright posture, which can lead to different biomechanical outcomes. Bumann et al. [41] demonstrated that greater pelvic rigidity during walking may represent a compensatory mechanism to keep the spinal canal more open during walking, hence reducing pain.

Body weight changes can also impact the biomechanics of the lumbar spine. Increased body weight can lead to higher compressive forces on the spine, exacerbating symptoms of LSS. Corazzelli et al. [42] found that an increase in body mass index (BMI) was often accompanied by a decrease in the cross-sectional area of the Erector Spinae muscles, which can contribute to greater disability in LSS patients. Abbas et al. (8) noted that vertebral morphometry, including vertebral body width and spinal canal diameters, is associated with the development of degenerative lumbar spinal stenosis, suggesting that body weight and vertebral dimensions play a role in symptomatology.

Muscle degeneration and spinal balance are critical in the pathophysiology of LSS. Han et al. highlighted the role of muscle degeneration and spinal balance in LSS, noting that muscle quality and fatigue can influence posture and ambulatory biomechanics [43]. Schönnagel et al. [44] found a significant association between LSS and axial muscle wasting, which could worsen LSS due to increased spinal instability. This study protocol aims to investigate these factors further to understand their impact on LSS symptoms and treatment outcomes, highlighting the need for further research investigating the association between axial muscle wasting and worsening LSS.

Cerebrospinal fluid dynamics also play a role in LSS. Chun et al. [45] conducted a pilot study to compare cerebrospinal fluid hydrodynamics at the lumbosacral spinal level between patients with spinal stenosis and healthy controls. The study found that cerebrospinal fluid circulation was impaired in patients with spinal stenosis. Specifically, the researchers found that CSF flow at the sacral level was barely detectable at the sacral level in patients with LSS. Additionally, the flow velocities were slower in patients with LSS compared to controls. These findings may contribute to both the pathophysiology and biomechanics of this condition [45].

Surgical interventions can significantly impact the biomechanics of the lumbar spine. Bresnahan et al. evaluated the biomechanical changes resulting from different surgical techniques for treating lumbar stenosis. They found that minimally invasive procedures that preserve posterior elements result in greater preservation of normal lumbar spine motion post-surgery compared to traditional laminectomy techniques [46]. This suggests that the surgical approach can significantly impact postoperative biomechanics and patient outcomes.

In summary, the biomechanical changes in patients with lumbar spinal stenosis involve complex interactions between spinal loading, pelvic tilt, body weight, gait, posture, muscle activity, cerebrospinal fluid dynamics, and surgical interventions. These factors collectively influence the severity and progression of symptoms in LSS patients. Understanding these biomechanical aspects can help in developing targeted interventions to alleviate symptoms and improve function in individuals with LSS.

### Spinal Stenosis Management

#### Nonsurgical/Conservative Treatment

##### Physical Therapy:

**Figure 3:**
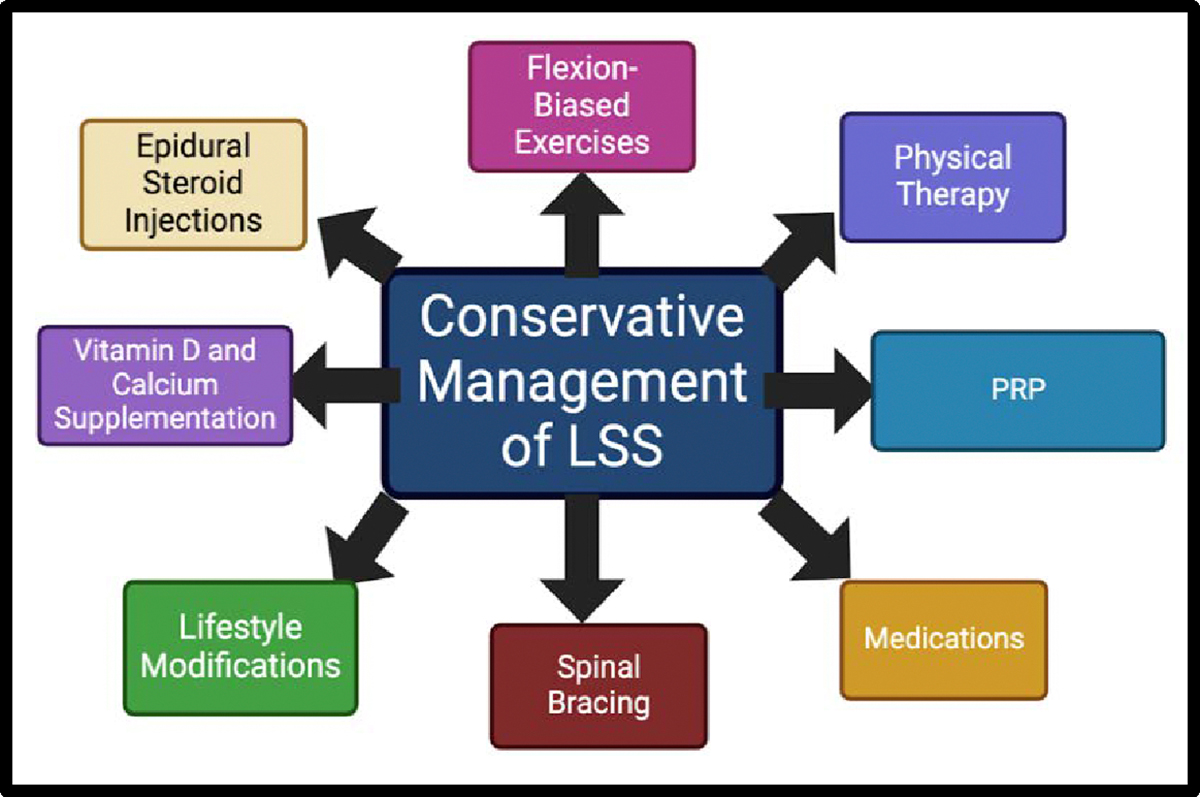
Conservative Management Options for lumbar spinal stenosis (LSS). PRP, platelet rich plasma.

Physical therapy is a mainstay treatment modality for lumbar spinal stenosis. This holds especially true for patients with contraindications to surgical correction. Therapy focuses on reducing pain and improving the overall function of the region. Pain relief modalities usually start with heat or ice therapy. Heat therapy will vasodilate the region to allow increased blood flow. This will provide more nutrients and oxygen to promote recovery. Heating the area will also relax tense muscles that could exacerbate the symptoms. Ice therapy will provide acute relief of symptoms by reducing inflammation. Cold stimulation will constrict blood vessels and decrease nervous system activity, reducing pain response to the region. Patients will apply ice or heat packs to the area for 15–20 minutes at a time to manage symptoms (Figure 3).

Physical therapists can implement a variety of musculoskeletal techniques to promote healing of the area. These techniques include but are not limited to joint mobilization, myofascial release, and soft tissue mobilization. A 2021 systematic review revealed that manual manipulative therapy techniques such as myofascial release may be effective in aiding patients post-surgery for chronic low back pain (47).

The review revealed that lumbar mobilization improved the range of motion in lumbar extension in patients post-L5 laminectomy. However, more studies and research are needed to arrive at a definite conclusion. The study reveals that physical therapy is a potentially viable treatment option for patients managing symptoms of low back pain (48).

Another study revealed that thoracic mobilization and soft tissue techniques were both viable in reducing unnecessary lumbar muscle activation, thus reducing chronic low back pain (49). Research is limited in exploring the effects of physical therapy on lumbar spinal stenosis. The results are promising and reveal it as an acceptable treatment modality for patients.

##### Exercise:

Exercise is an important aspect for patients suffering from lumbar spinal stenosis. Stretching and strength training of muscles in the area can help to decrease biomechanical stress. Through improving posture and decreasing weakness of the musculature, patients can provide themselves with markedly significant symptomatic relief (Figure 3). Treatment involves dedicated programs that patients will implement into their day-to-day routines. Stretching exercises include pelvic tilt, sit-up in knee flexion, double knees to chest to improve function of the erector spinae, seated flexion, and strengthening of quadriceps and gluteus maximus muscles, All of these muscles have attachments or indirect effect on the lumbar region and provide pain relief [50]. There are numerous articles discussing the efficacy of stretch exercises on lumbar spinal stenosis. A systematic review revealed that exercise was effective in reducing pain, need for analgesics, disability, and even mood disturbances in patients with lumbar spinal stenosis [51].

Another study revealed that given an at-home exercise program, patients with lumbar spinal stenosis effectively reduced their pain and reported lower scores in self-answered questionnaires. The program included the exercises knee-to-chest exercises, thoracic extension self-mobilization, double knee-to-chest exercises, lower abdominal strengthening exercises, lumbar rotation stretching, hip abduction strengthening exercises, rectus femoris self-stretching, and iliopsoas self-stretching. While stretching improved pain, it did not have a significant effect on improving gait [52].

A study indicated that when compared to strength training, core stabilization exercises reduced the symptoms of non-specific low back pain with greater efficacy. This study highlights the versatility of training programs that can be used in the treatment of lumbar spinal stenosis (53).

##### Pharmacology:

Pharmacological options are crucial in treating lumbar spinal stenosis. Medications can provide immediate relief from moderate to severe symptoms and greatly improve the quality of life for patients (Figure 3). Initial pharmacological therapy involves the use of analgesics such as NSAIDs for immediate symptomatic relief. Analgesics coupled with physical therapy and activity modification are the primary conservative treatment protocol for patients with lumbar spinal stenosis. In one study utilizing this treatment regimen, approximately 1/3 of patients reported improvement of symptoms, 50% reported no change in symptoms, and about 10–20% reported worsening symptoms. This study shows that conservative treatment has mixed results at addressing the symptoms of lumbar spinal stenosis [1].

Should patients be refractory to analgesic treatment, physicians can opt to treat them with epidural steroid injections. The goal of steroid injection is to reduce inflammation, which should improve symptoms and quality of life. There are two different types of injections: particulate and non-particulate. Particulate injections refer to steroids with shorter half-lives but significantly faster onset. Examples of particulate injections include methylprednisolone acetate and triamcinolone. Non-particulate injections refer to steroids with longer half-lives but slower onset. An example of a non-particulate injection includes dexamethasone. The injection can be performed in 3 different positions - interlaminar, transforaminal, or caudal. The interlaminar approach is the most frequently used. A review of the current literature reveals that steroid injections are effective in reducing pain, however, the effects are not long-lasting [54].

##### Assisted Devices:

Physicians may also choose to use assisted devices to help alleviate symptoms of lumbar spinal stenosis, such as bracing and spinal supports. However, research is limited, and not enough evidence is available to support the use of spinal supports. One study looked at the use of corset use to improve strength and posture of the lumbar spine. In a study of 40 patients, the use of corsets improved short-term relief of low back pain and strengthening of paravertebral muscles. The study concluded that corsets may be an effective approach to treating low back pain [55]. Another study showed that the use of lumbar belts could help reduce the need for pharmacologic intervention and improve functional status in patients [56]. An overall meta-analysis of the use of supports revealed that assisted devices offer definite improvement in disability, however data was insufficient in determining their effectiveness in subacute or recurrent back pain. The data shows that spinal supports offer short-term benefits, but their long-term benefits still need to be further investigated [57].

#### Challenges and Success in Conservative Management

Given the research, conservative management can be an effective method in treating lumbar spinal stenosis. Management primarily revolves around treating the symptoms and improving the function of the lumbar spine. This should ultimately help to reduce severe symptomatic flairs and improve the quality of life for patients suffering from lumbar spinal stenosis. Challenges to recovery include a strict adherence to treatment regimens. This can prove to be markedly challenging for patients, as therapy and exercise will often exaggerate the pain. If conservative management fails, then patients can choose to seek surgical intervention, which has been proven to be a highly effective option.

#### Surgical Treatments for Lumbar Stenosis

**Figure 4:**
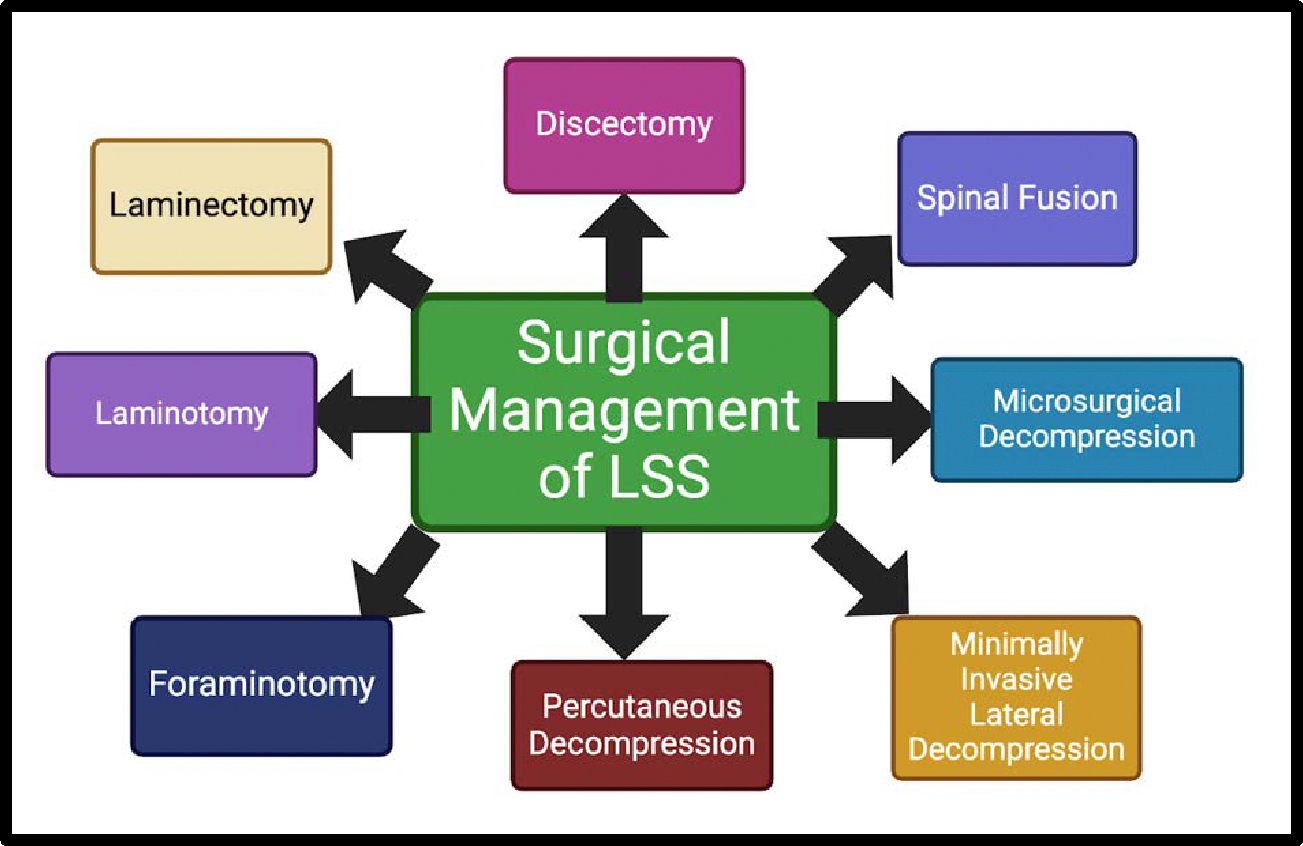
Surgical Management Options for lumbar spinal stenosis (LSS).

Patients with spinal stenosis undergo surgery electively unless the patient is experiencing a rare emergent condition such as cauda equina syndrome. Patients will be monitored since the onset of LSS and considered for surgical repair if the pain becomes persistent, refractory, or progressively worsening despite the use of maximal conservative care for 3 to 6 months or if the neurological functions begin to deteriorate [58]. Various surgical management options for LSS are shown in Figure 4.

The most performed surgeries are intended to decompress the nerves within the spinal cord. There are direct and indirect procedures that can be implemented to achieve decompression of the spine; direct requiring the visualization of the dural sac during surgery, while indirect being done without the need for visualization of the dural sac as the goal is not to resect any compressing tissues [59]. Among the most performed direct surgical procedures for lumbar stenosis are the conventional laminectomy (gold standard) or lumbar laminotomy with discectomy for disc herniations, as well as minimally invasive procedures such as partial facetectomy and split-spinous process laminoplasty [60] (Figure 4).

#### Indirect Treatment methods

Indirect treatments for lumbar spinal stenosis aim to alleviate symptoms without directly decompressing the spinal canal. One such technique is interbody fusion, which can be performed using various approaches including posterior lumbar interbody fusion (PLIF), transforaminal lumbar interbody fusion (TLIF), oblique lumbar interbody fusion (OLIF), and lateral lumbar interbody fusion (LLIF). The literature demonstrates that interbody fusion is indicated in the presence of spinal instability, degenerative spondylolisthesis, or deformity such as scoliosis. However, Yone et al. [61] demonstrates that interbody fusion provides stability and prevents further degeneration but is associated with longer operative times and increased blood loss.

The literature also discusses cutting-edge minimally invasive lumbar decompression procedures (MILD) which aim to relieve pressure on the spinal cord while allowing for minimal disruption of surrounding tissue [62]. This study discusses a minimally invasive treatment of degenerative central canal LSS with ligamentum flavum hypertrophy through percutaneous decompression of the hypertrophic ligamentum flavum. This procedure involves excision of the interlaminar bone and ligamentum flavum that are causing the narrowing of the spinal canal. The most common approach is the percutaneous dorsal approach which involves increasing space in the spinal canal with minimal disruption to the surrounding tissue [62]. Patients that are optimal candidates for this procedure are symptomatic LSS patients with central canal narrowing and ligamentum flavum hypertrophy confirmed on MRI with symptoms of neurogenic claudication [62].

#### Conventional laminectomy with and without fusion

The conventional laminectomy aimed at posterior spinal decompression is among the most performed spinal surgeries in patients over 65 years of age [63]. It is most useful to alleviate patients with degenerative stenosis, fractures, primary and secondary spinal tumors, abscesses, and deformities of the spine. The procedure is aimed at removing the spinous process and lamina, limited to the medial part of the facet joint, and all components that could be obstructing the neurological structure including the central canal, the lateral recess, and the neural foramina which are collectively decompressed to avoid failed back surgery [64].

A less invasive alternative technique is the laminotomy with or without discectomy, which involves removing only a portion of the lamina to decompress the affected area as well as shave off any aggravating disc protrusions present. No statistically significant clinical improvements are seen using this similar technique in comparison to a laminectomy for treating spinal stenosis, but this procedure is indicated for patients with severe disc herniations who require decompression [65].

There are many different surgical techniques for laminectomy with the most common being the classic open approach. A posterior midline incision is made (3 to 4 cm) and the paraspinous muscles are dissected and retracted from the spinous process attachments to avoid facet joint damage. The spinous process and dorsal laminae may then be resected with the bone cutting rongeur or burr, and the ligamentum flavum that is now exposed can be resected with the Woodson elevator and spatula. Medial facetectomies can also be performed to decompress the lateral recess and greater decompression can be achieved in the foraminal region using the Kerrison rongeurs. The ball tip and angled probe is then used to assess the foraminal size, but great care must be taken to ensure no damage is made to the pars interarticularis and the facet joint itself which may risk potential spinal instability and post-surgical complications. The final step is confirmation that the dural sac is exiting and extending with the nerve roots.

The most controversial question lies in whether the laminectomy should be done with a fusion. According to Fischgrund et al. [66], the indication for spinal fusions would be any spinal instability, degenerative or isthmic spondylolisthesis, kyphosis, trauma, tumors, infections, neuroforaminal stenosis with compressed exiting nerves, or scoliosis, as laminectomy done alone can increase the risk of worsened spinal instability in these patients [66].

The surgical outcomes of laminectomies with and without instrumented fusion have been studied and compared using the Medical Outcomes Study 36-item short-form health survey (SF-36) and the Oswestry disability index (ODI). Higher scores on the ODI indicated increased disability related to back pain while higher scores on the SF-36 indicated a better quality of life for patients. study physical component scores for patients 2, 3, and 4 years postoperatively. The SF-36 scores were significantly higher in patients with the fusion after all post-operative years. ODI scores, however, had no statistically significant difference in comparison for the two patient populations. Patients who underwent the laminectomy with fusion also had lower rates of reoperation over the 4 years post-op at 14% in comparison to those without the fusion at 34%. Although Laminectomies with fusion benefit patient quality of life and lower reoperations, there are significantly higher rates of bleeding, longer hospital stays, and longer duration of operations. With these complications related to the operation, the laminectomy with fusion must be reserved for candidates without significant morbidities and elderly people [67].

### Minimally Invasive Techniques

#### Lumbar spinous process-splitting laminoplasty (LSPSL)

A more recent and less invasive technique for decompression called lumbar spinous process-splitting laminoplasty (LSPSL), involves splitting the spinous process longitudinally and then dividing the base from the posterior arch while leaving the paraspinal muscles bilaterally attached to the lateral portion. This procedure is done with the hope of maintaining the integrity of the supporting paraspinal muscles to result in a quicker recovery process.

The procedure requires obtaining a templating for the spinous process, measuring the patients spinous process AP axis as well as the shortest depth in hopes of avoiding overpenetration injuries to the spine. An incision is made midline to the spinous process at the level of stenosis and dissected to the thoracolumbar fascia. The dorsal part of the bone is decorticated using a 2-mm round burr and a #15 blade is used to incise the supraspinous and interspinous ligaments in a line. The spinous process can then be split using a thin osteotome and is detached from the base of determined depth while the muscular attachments to the spinous process remain intact. A self-retaining retractor will hold the two halves in place while there is room to perform the decompression, using a microscopic or loupe magnification. The procedure requires removal of the superior lamina, proximal aspect of the inferior lamina, ligamentum flavum, and undercutting the facet on both sides through the midline. Ad rain can be placed, and the spilt bone products can be rejoined using the transosseous restorable sutures [68].

A study was done to compare the surgical outcomes for patients who underwent conventional laminectomies and modified LSPSL, where a laminoplasty was done instead of a laminectomy, using the Japanese Orthopedic Association (JOA), a clinical symptom scale. The recovery rate of LSPSL was 64.2% while conventional laminectomy was 68.2%. Twelve months after surgery the patients with conventional laminectomies had a greater degree of paraspinal muscle atrophy at 22.2% while the degree of LSPSL paraspinal muscle atrophy was 7.8% [69]. The atrophy decrease was improved compared to traditional laminectomy 1 month postoperatively using axial MRIs [70].

Though there is some support for adopting a less invasive approach with nuanced techniques, other researchers like Rajasekaran et al. [71], have shown in their randomized controlled trials that there is no difference between the groups in number of decompressed levels, operative time, intraoperative blood loss, and length of hospital stay. Comparing outcomes for up to a year, there was no difference in outcomes using the JOA score, neurogenic claudication outcome score, VAS (visual analogue scale) for back pain, and VAS for neurogenic claudication [71].

#### Partial facetectomy

Partial facetectomy is a surgical procedure often performed for patients with lumbar spinal stenosis, particularly those without instability or central canal stenosis. This technique is specifically indicated for individuals with isolated foraminal stenosis and lumbar radiculopathy that has not responded to nonoperative treatments. A combined transarticular lateral and medial approach with partial facetectomy is the preferred operative technique due to the lower rates of secondary spinal instability when compared to the complete facetectomy according to the literature [72].

The common surgical approach for this procedure is the paramedian (Wiltse) approach, which is effective for both unilateral and bilateral disease. In cases of bilateral disease, two incisions are made. This approach offers a 45-degree angled view of the facet joints, providing excellent access while minimizing muscle damage through meticulous dissection between muscle bellies. The facet joint is incised at its superior aspect, and the pedicle is carefully identified using a dental instrument to prevent injury. A stiletto osteotome is then used to osteotomize the facet, ensuring nerve root protection throughout the process. Hemostasis is achieved with bipolar cautery, and the foramen is enlarged using a Kerrison rongeur to remove any protruding edges or redundant ligamentum flavum [73].

In terms of recovery, Kang et al. [73] retrospectively analyzed 48 patients who underwent partial facetectomy for foraminal stenosis between 2001 and 2010. Of the 47 patients included in the study, 28 were on disability for durations ranging from 2 to 28 months due to muscle weakness without atrophy and diminished reflexes.

At an average follow-up of 3.8 years, outcomes were encouraging for most patients. Twenty-seven patients reported no back pain and returned to normal activity levels. Eight patients experienced occasional moderate back pain that did not require analgesics, while six patients showed signs of further degeneration after an average of 5.6 years. Five patients required a second surgery for additional decompression [73].

The post-operative advantages of a partial facetectomy, particularly when using the Wiltse approach, are well-documented in the literature. One significant advantage includes the preservation of spinal stability. According to Hejazi et al. [72], the combined transarticular lateral and medial approach with partial facetectomy allows for decompression of the intervertebral foramen while maintaining facet joint integrity. This minimizes the risk of secondary instability that can be caused by a complete facetectomy [72].

#### Surgical vs Nonsurgical Treatment Modality

The Spine Patient Outcome Research Trial (SPORT), one of the biggest trials done to date, performed a randomized control and cohort study on patients with lumbar stenosis enrolled from March 2000 to November 2004 from 13 multidisciplinary spine clinics in 11 states who underwent surgical and non-surgical procedures for back and leg symptoms [74]. The patients in the observational cohort group, who chose elective surgical intervention reported greater improvements than those who preferred a nonoperative route, but the outcomes being self-reported were potentially subjected to confounding error and should be cautiously valued [74]. In the randomized controlled trial, the surgery and nonoperative treatment groups improved substantially over a 2-year period, but because of the crossover in both directions, the conclusion to determine superiority or equivalence of the treatment groups was not warranted because of the intent-to-treat [75]. In an as-treated analysis of the combined randomized and observational trials of symptomatic spinal stenosis, the ones treated surgically over conservative management showed a greater improvement in pain, function, satisfaction, and self-related progress over 2 years compared to those treated conservatively and these results were supported for up to 4 years of analysis [76].

Several other studies have investigated patient outcomes in surgical vs conservative care, with one low-quality evidence from a small study revealed no difference in pain outcomes between decompression and usual conservative care (bracing and exercise) at three months, four years, and 10 years [77]. In addition, the decompression and conservative treatments had similar results for disability using the Oswestry Disability Index (ODI) at three, six, and 12 months, but one study reported greater improvements for surgical decompression [77,78]. Small trial studies also have shown no difference between steroidal epidural injections and mild decompression procedures, but these were considered low-quality evidence [79]. Another single study showed spinal surgical decompression with interspinous spacers were favorable over conservative measures at 6 weeks, 6 months, and one year follow-up for symptom severity and physical function [80].

In a meta-analysis done by Zaina et al. [81] which included the SPORT trial, there was no significant differences in the Oswestry Disability Index at six months and at one year when comparing direct decompression with or without fusion versus a multi-modal non-operative care plan, but a difference favoring decompression at 24 months. However, the 24-month improvement was based on the low-quality evidence performed on 320 participants [81]. Longer follow-up dates were not available to cross reference outcomes in these studies and require further investigation in future studies.

Factors to consider when electing to undergo surgical management are the complications associated with surgery. Weinstein and colleagues [78] reported a 10% rate of perioperative complications and 10% postoperative, Zucherman and co-investigators [80] reported a combined rate of 11% perioperative and postoperative side effects, and Malmivaara and co-investigators [82] reported a side effect rate of 24%. The side effect profile in these three studies widely ranged from 10% to 24%, which included spinous process fracture, coronary ischemia, respiratory distress, hematoma, stroke, risk of reoperation and death due to pulmonary edema in these three studies makes it imperative that the risks are discussed with patients prior to going through any elective surgical interventions. Because there is no clear-cut evidence supporting surgeries are the better intervention measure, it is important to look at patient risk factors and comorbidities in treatment selection.

#### Comorbidities to Consider in Treatment Direction

Lumbar spinal stenosis (LSS) is a common condition, particularly in aging populations, often requiring surgical intervention for symptom relief. However, various comorbidities significantly impact surgical outcomes, complications, and recovery. Diabetes mellitus (DM) has been shown to be a major factor associated with poorer prognosis and increased medical costs in patients undergoing LSS surgery. A nationwide study by Lee et al. (83) compared outcomes between a DM group (n=3,478) and a non-DM group (n=10,820), demonstrating higher admission rates, medical costs, and worse survival rates among diabetic patients, especially those with chronic kidney disease (CKD). Despite these challenges, surgical intervention still provided better outcomes for DM patients compared to conservative treatment, emphasizing the relative benefits of decompression surgery in this population. Additionally, insulin-dependent DM has been identified as a stronger predictor of major complications compared to diabetes managed with diet or oral agents, emphasizing the importance of glycemic control prior to surgery [84].

Obesity, particularly a body mass index (BMI) greater than 30, also plays a significant role in poor postoperative outcomes. Papavero et al. [85] identified BMI as a negative prognostic factor due to the challenges of impaired visualization during surgery, slower rehabilitation, and accelerated degenerative changes. Despite these difficulties, obese patients reported sufficient improvement postoperatively to justify the surgery. Addressing obesity preoperatively could improve visualization during decompression and promote faster recovery. Similarly, preoperative opioid use has been linked to lower odds of significant improvement postoperatively. Weiner et al. [86] highlighted the detrimental impact of chronic opioid use, which likely stems from opioid tolerance and associated psychosocial factors. Clinicians should consider alternative pain management strategies to mitigate these risks and improve outcomes.

Mental health also profoundly influences recovery. Sinikallio et al. [87] demonstrated that patients with continuous depression experienced poorer improvements in symptom severity, disability scores, and walking capacity following surgery. Notably, patients who recovered from depression postoperatively showed outcomes comparable to those without any mood disturbances, highlighting the importance of addressing mental health preoperatively. Age, particularly in conjunction with comorbidities, further compounds the risk of complications. Studies by Li et al. [88] and Raffo and Lauerman [89] revealed that patients over 80 with multiple comorbidities had significantly higher complication and mortality rates compared to younger, healthier cohorts [85,86]. For example, those over 85 with three or more comorbidities had an 18.9% complication rate, compared to 6% in younger patients. However, a systematic review by Liang et al. [90] found that clinical improvement in pain and disability did not significantly differ by age, suggesting that advanced age alone should not be considered a contraindication for surgery when appropriately managed.

Additional predictors of complications include cardiovascular disease (CVD), hypertension, chronic steroid use, and functional status, with smoking and alcohol use being less impactful [84]. These findings emphasize the importance of thorough preoperative assessments and optimization of comorbidities, particularly in patients with DM, obesity, or advanced age. By addressing modifiable factors like glycemic control, mental health, and opioid dependence, surgeons can improve overall outcomes and reduce complication rates. While high-risk populations face greater challenges, the clinical benefits of decompression surgery for lumbar spinal stenosis remain evident, particularly when appropriate preoperative risk stratification is applied.

### Conclusion and Future Directions

Lumbar spinal stenosis (LSS) is a prevalent condition that significantly affects daily activities of the patients due to the debilitating physical and neurogenic symptoms, which interfere with basic functions like walking and standing. In 2020, research pooling multiple studies estimated the prevalence of LSS with clinical diagnostic criteria to be approximately 11% in the general population [91]. However, the diagnostic criteria for LSS are not universally agreed upon and vary across different guidelines due to differences in symptom presentation, disease progression, and diagnostic methodologies. Despite these variations, Magnetic Resonance Imaging (MRI) is widely regarded as the most valuable and reliable imaging modality for diagnosing LSS, offering detailed visualization of the spinal canal, nerve roots, and any structures that may be contributing to nerve compression.

After several years of different diagnostic and treatment modalities used for patients, there is still uncertainty in the efficacy of deciding whether to treat patients conservatively or nonconservatively. To gauge the effectiveness of the treatment modalities, researchers have compared patients’ quality of life post-op and the need for repeat surgery after the initial one. In one systematic review, the outcomes of surgical treatments such as spinal decompression or interspinous device implants showed significantly greater improvements in regard to pain relief, quality of life, and disability reduction within the 3–6-month post-op time period. However, after 2–4 years, the difference between the outcomes for surgical vs non-surgical (medications, physical therapy, or injections) outcomes diminishes over time. The outcomes of this study indicate the short-term benefit of surgical intervention, while the long-term benefit is less clear [92].

Comorbidities are factors that must be considered when trying to make a decision on preceding with treatment for LSS whether conservatively or non-conservatively. A systematic review looked at several comorbidities and their impact on the treatment outcomes of patients with symptomatic LSS. This analysis incorporates data from 51 studies, focusing on key outcomes such as patient satisfaction, functional improvements, symptom relief, and the occurrence of adverse events (AEs) in patients who have comorbidities such as cardiovascular disease, diabetes, obesity, smoking, etc. The findings suggest that, while comorbidities do not significantly reduce patient satisfaction compared to individuals without comorbidities, they do increase the risk of AEs. Specifically, patients with diabetes were found to have a higher likelihood of experiencing adverse events. The study also revealed that older age did not significantly affect patient satisfaction, symptom alleviation, or functional improvements [93].

There are many innovations in the surgical intervention of LSS that are increasingly being used and extensively researched for effectiveness and safety. Stem cell research, like in many other fields, is a relatively new and undiscovered style of treatment for cancers, biomechanical repairs, stroke recovery, heart repairs, etc. A double-blind control experiment on Intravertebral disc repair using allogenic bone marrow-derived mesenchymal stem cells after discectomy for patients with LSS. The study, currently being conducted, is investigating the safety and efficacy of this post-surgical technique by looking at patients’ post-operative pain and degenerative progression [94].

Other innovations in the surgical interventions/implants for LSS that are currently being studied include the total posterior spine system (TOPS). The TOPS is a mechanical implant replacing a whole vertebra that innovates both stability and motion to the spinal segment, unlike the traditional laminectomy with fusion that sacrifices motion for stability. Another innovation that is less invasive than TOPS is the Vertiflex superion interspinous spacer which is placed through a small incision with fluoroscopic imaging guidance placed in between the spinous processes [95]. This indirectly decompresses the spinal nerves by lengthening the space in which the spinal nerves exit the canal. The TOPS and vertiflex devices have been studied in short-term, and they were discovered to have been both safe and efficacious in the treatment of LSS [96,97]. With the increased surgical advancements used for the treatment of LSS, there are still many long-term studies that must be done to fully understand their effects on patients.

## Key Points:

- Lumbar spinal stenosis (LSS) is the narrowing of the spinal canal, causing neural and vascular compression, leading to symptoms like neurogenic claudication and lower extremity weakness.
- LSS affects over 103 million people globally and more than 200,000 people annually in the U.S., primarily older adults.
- Degenerative changes, including ligamentum flavum hypertrophy, intervertebral disc degeneration, and facet joint osteoarthritis, are the leading causes of acquired LSS.
- Early-onset LSS results from shorter pedicle lengths and smaller spinal canal dimensions, often affecting multiple levels with fewer degenerative changes.
- Osteoarthritis, increased BMI, and other degenerative conditions commonly coexist with LSS, compounding symptoms.
- Rheumatoid arthritis and ankylosing spondylitis are rare causes of LSS, with RA typically affecting the cervical spine but occasionally seen in lumbar cases.
- Restricted blood flow and venous congestion in the spinal canal contribute to neurogenic claudication, a hallmark of LSS symptoms.
- MRI is the gold standard for diagnosing LSS, but a thorough clinical examination is essential due to overlapping symptoms with other conditions.
- Forward flexion alleviates LSS symptoms by widening the spinal canal, while spinal extension exacerbates pain by narrowing it.
- Non-surgical options include physical therapy, pharmacologic treatments (e.g., NSAIDs, steroid injections), and lifestyle changes to alleviate symptoms.
- Laminectomy remains the gold standard, while minimally invasive techniques, such as interspinous devices and ligamentum flavum decompression, are promising alternatives (Lurie et al., 2016).
- Research into stem cell therapy, genetic predispositions, and innovative surgical techniques aims to improve long-term outcomes for patients with LS.

## References

[1] Katz JN, Zimmerman ZE, Mass H, Diagnosis and Management of Lumbar Spinal Stenosis: A Review. JAMA. 2022;327(17): 1688–1699. 35503342 10.1001/jama.2022.5921

[2] Ravindra VM, Senglaub SS, Rattani A, Degenerative lumbar spine disease: estimating global incidence and worldwide volume. Global Spine J. 2018;8(8): 784–794. 30560029 10.1177/2192568218770769 PMC6293435

[3] Lurie J, Tomkins-Lane C. Management of lumbar spinal stenosis. BMJ 352 (2016): h6234. 26727925 10.1136/bmj.h6234 PMC6887476

[4] Ammendolia C, Cote P, Ramersaud YR, The Boot Camp Program for Lumbar Spinal Stenosis: a protocol for a randomized controlled trial. Chiropr Man Therap 24 (2016): 25–40. 10.1186/s12998-016-0106-y PMC4948101 27433335

[5] Kalichman L, Cole R, Kim DH, Spinal stenosis prevalence and association with symptoms: The Framingham Study. Spine J 97 (2009): 545–550. 10.1016/j.spinee.2009.03.005 PMC3775665 19398386

[6] Young JJ, Jensen RK, Hartvigsen J, Prevalence of multimorbid degenerative lumbar spinal stenosis with knee or hip osteoarthritis: a systematic review and meta-analysis. BMC Musculoskelet Disord 23 (2022): 177. 35209884 10.1186/s12891-022-05104-3 PMC8876450

[7] Abbas J, Hamoud K, May H, Socioeconomic and physical characteristics of individuals with degenerative lumbar spinal stenosis. Spine 38 (2013): E554–561. 24477055 10.1097/BRS.0b013e31828a2846

[8] Abbas J, Peled N, Hershkovitz I, The Role of Vertebral Morphometry in the Pathogenesis of Degenerative Lumbar Spinal Stenosis. Biomed Res Int 4 (2021): 7093745. 10.1155/2021/7093745 PMC8437646 34527742

[9] Kawaguchi Y, Matsuno H, Kanamori M, Radiologic findings of the lumbar spine in patients with rheumatoid arthritis, and a review of pathologic mechanisms. J Spinal Disord Tech 16 (2003): 38–43. 12571483 10.1097/00024720-200302000-00007

[10] Luken Martin G III. Patel, Ellman Dushyant V., Michael H. Symptomatic Spinal Stenosis Associated with Ankylosing Spondylitis. Neurosurgery 11 (1982): p703–705. 10.1227/00006123-198211000-00017 7155336

[11] Singh K, Samartzis D, Vaccaro AR, Congenital lumbar spinal stenosis: a prospective, control-matched, cohort radiographic analysis. Spine J 5 (2005): 615–622. 16291100 10.1016/j.spinee.2005.05.385

[12] Katz JN, and Harris MB Lumbar spinal stenosis. New England Journal of Medicine 358 (2008): 818–825. 18287604 10.1056/NEJMcp0708097

[13] Genevay S, and Atlas SJ Lumbar spinal stenosis. Best Practice & Research Clinical Rheumatology 24 (2010), 253–265. 20227646 10.1016/j.berh.2009.11.001 PMC2841052

[14] Atlas SJ, Keller RB, Robson D, Surgical and nonsurgical management of lumbar spinal stenosis: four-year outcomes from the Maine lumbar spine study. Spine (Phila Pa 1976) 25 (2000): 556–562. 10749631 10.1097/00007632-200003010-00005

[15] Lee BH, Moon SH, Suk KS, Lumbar Spinal Stenosis: Pathophysiology and Treatment Principle: A Narrative Review. Asian Spine J. 2020 Oct; 14 (2020): 682–693. 33108834 10.31616/asj.2020.0472 PMC7595829

[16] Aleksić V, Todorović J, Miladinović N Ligamentum flavum analysis in patients with lumbar discus hernia and lumbar spinal stenosis. Sci Rep 13 (2023): 3804. 36882487 10.1038/s41598-023-30928-x PMC9992359

[17] Xu J, Si H, Zeng Y, Transcriptome-wide association study reveals candidate causal genes for lumbar spinal stenosis. Bone Joint Res 12 (2023): 387–396. 37356815 10.1302/2046-3758.126.BJR-2022-0160.R1 PMC10290907

[18] Shi H, Li S, Liu S, Facet joint tropism, pelvic incidence and intervertebral height index: associations with facet joint osteoarthritis in lumbar spinal stenosis. Spine J 24 (2024): 317–324. 37844628 10.1016/j.spinee.2023.10.001

[19] Ma Y, Huang P, Tu Z Associations between facet tropism and vertebral rotation in patients with degenerative lumbar disease. Eur J Med Res 26 (2021): 149. 34930499 10.1186/s40001-021-00622-7 PMC8686366

[20] Nakamura T, Okada T, Endo M Angiopoietin-like protein 2 promotes inflammatory conditions in the ligamentum flavum in the pathogenesis of lumbar spinal canal stenosis by activating interleukin-6 expression. Eur Spine J 24 (2015): 2001–2009. 25735609 10.1007/s00586-015-3835-z

[21] Sugimoto K, Nakamura T, Tokunaga T, Angiopoietin-Like Protein 2 Induces Synovial Inflammation in the Facet Joint Leading to Degenerative Changes via Interleukin-6 Secretion. Asian Spine J. 2019 Jun;13(3): 368–376. 30685956 10.31616/asj.2018.0178 PMC6547404

[22] Lai MKL, Cheung PWH, Cheung JPY. A systematic review of developmental lumbar spinal stenosis. Eur Spine J 29 (2020): 2173–2187. 32623513 10.1007/s00586-020-06524-2

[23] Lai MKL, Cheung PWH, Song YQ, Pedigree analysis of lumbar developmental spinal stenosis: Determination of potential inheritance patterns. J Orthop Res 39 (2021): 1763–1776. 32902878 10.1002/jor.24850

[24] Akar E, Somay H. Comparative morphometric analysis of congenital and acquired lumbar spinal stenosis. J Clin Neurosci 68 (2019): 256–261. 31331753 10.1016/j.jocn.2019.07.015

[25] Byvaltsev VA, Kalinin AA, Hernandez PA, Molecular and Genetic Mechanisms of Spinal Stenosis Formation: Systematic Review. Int J Mol Sci 23 (2022): 13479. 36362274 10.3390/ijms232113479 PMC9658491

[26] Schroeder GD, Kurd MF, & Vaccaro AR Lumbar spinal stenosis: how is it classified? JAAOS-Journal of the American Academy of Orthopaedic Surgeons 24 (2016): 843–852. 10.5435/JAAOS-D-15-00034 27849674

[27] Hall S, Bartleson JD, Onofrio BM, Lumbar spinal stenosis: clinical features, diagnostic procedures, and results of surgical treatment in 68 patients. Annals of internal medicine 103 (1985): 271–275. 3160275 10.7326/0003-4819-103-2-271

[28] Suri P, Rainville J, Kalichman L, Does this older adult with lower extremity pain have the clinical syndrome of lumbar spinal stenosis? JAMA 304 (2010): 2628–2636. 21156951 10.1001/jama.2010.1833 PMC3260477

[29] Haig Andrew J. Diagnosis and Management of Lumbar Spinal Stenosis. JAMA 303 (2010), 71. 20051574 10.1001/jama.2009.1946

[30] Munakomi S, Foris LA, Varacallo M. Spinal Stenosis and Neurogenic Claudication. [Updated 2023 Aug 13]. In: StatPearls [Internet]. Treasure Island (FL): StatPearls Publishing; 2024 Jan-. Available from: https://www.ncbi.nlm.nih.gov/books/NBK430872/. 28613622

[31] Chagnas MO., Poiraudeau S, Lefèvre-Colau MM Diagnosis and management of lumbar spinal stenosis in primary care in France: a survey of general practitioners. BMC Musculoskelet Disord 20 (2019): 431. 31521138 10.1186/s12891-019-2782-y PMC6745066

[32] Munakomi S, Kumar BM. Wasting of Extensor Digitorum Brevis as a Decisive Preoperative Clinical Indicator of Lumbar Canal Stenosis: A Single-center Prospective Cohort Study. Ann Med Health Sci Res 6 (2016): 296–300. 28503347 10.4103/amhsr.amhsr_392_15 PMC5414442

[33] Fraser S, Roberts L, Murphy E. Cauda equina syndrome: a literature review of its definition and clinical presentation. Arch Phys Med Rehabil 90 (2009): 1964–1968. 19887225 10.1016/j.apmr.2009.03.021

[34] Comer Christine, Finucane Laura, Mercer Chris, SHADES of grey – The challenge of ‘grumbling’ cauda equina symptoms in older adults with lumbar spinal stenosis, Musculoskeletal Science and Practice Volume 45 (2020): 2468–7812. 10.1016/j.msksp.2019.102049 31439453

[35] Das JM, Dua A, Nadi M. Straight Leg Raise Test (Lasegue sign) [Updated 2024 Oct 6]. In: StatPearls [Internet]. Treasure Island (FL): StatPearls Publishing; (2024). 31424883

[36] Mousavi SJ, Lynch AC, Allaire BT, Walking Biomechanics and Spine Loading in Patients With Symptomatic Lumbar Spinal Stenosis. Front Bioeng Biotechnol 9 (2021): 751155. 34869263 10.3389/fbioe.2021.751155 PMC8636982

[37] Penning L Functional pathology of lumbar spinal stenosis. Clinical Biomechanics (Bristol, Avon) 7 (1992), 3–17. 23915611 10.1016/0268-0033(92)90002-L

[38] Kuwahara W, Kurumadani H, Tanaka N, Correlation between spinal and pelvic movements during gait and aggravation of low back pain by gait loading in lumbar spinal stenosis patients. Journal of Orthopaedic Science: Official Journal of the Japanese Orthopaedic Association 24 (2019): 207–213. 30322623 10.1016/j.jos.2018.09.002

[39] Perring J, Mobbs R, & Betteridge C Analysis of patterns of gait deterioration in patients with lumbar spinal stenosis. World Neurosurgery 141 (2020): e55–e59. 32387784 10.1016/j.wneu.2020.04.146

[40] Igawa T, Katsuhira J, Hosaka A, Kinetic and kinematic variables affecting trunk flexion during level walking in patients with lumbar spinal stenosis. PLOS ONE 13 (2018): e0197228. 29746537 10.1371/journal.pone.0197228 PMC5944950

[41] Bumann H, Nüesch C, Loske S, Severity of degenerative lumbar spinal stenosis affects pelvic rigidity during walking. The Spine Journal: Official Journal of the North American Spine Society 20 (2020): 112–120. 31479778 10.1016/j.spinee.2019.08.016

[42] Wang J, Ullah S, Solano MA, Changes in kinematics, kinetics, and muscle activity in patients with lumbar spinal stenosis during gait: A systematic review. The Spine Journal: Official Journal of the North American Spine Society 22 (2022): 157–167. 34116219 10.1016/j.spinee.2021.06.003

[43] Huang C, Ye J, Song Y, The effects of fatigue on the lower limb biomechanics of amateur athletes during a Y-balance test. Healthcare (Basel) 11 (2023): 2565. 37761762 10.3390/healthcare11182565 PMC10530907

[44] Schönnagel L, Zhu J, Camino-Willhuber G, Relationship between lumbar spinal stenosis and axial muscle wasting. The Spine Journal: Official Journal of the North American Spine Society 24 (2024), 231–238. 37788745 10.1016/j.spinee.2023.09.020

[45] Chun SW, Lee HJ, Nam KH, Cerebrospinal fluid dynamics at the lumbosacral level in patients with spinal stenosis: A pilot study. Journal of Orthopaedic Research: Official Publication of the Orthopaedic Research Society 35 (2017): 104–112. 27664416 10.1002/jor.23448

[46] Bresnahan L, Ogden AT, Natarajan RN, A biomechanical evaluation of graded posterior element removal for treatment of lumbar stenosis: Comparison of a minimally invasive approach with two standard laminectomy techniques. Spine 34 (2009): 17–23. 19127157 10.1097/BRS.0b013e318191438b

[47] Daniels CJ, Cupler ZA, Gliedt JA, Manipulative and manual therapies in the management of patients with prior lumbar surgery: A systematic review. Complement Ther Clin Pract 42 (2021): 101261. 33276229 10.1016/j.ctcp.2020.101261

[48] Timm KE A randomized-control study of active and passive treatments for chronic low back pain following L5 laminectomy. Journal of Orthopaedic & Sports Physical Therapy 20 (1994): 276–286. 7849747 10.2519/jospt.1994.20.6.276

[49] Lin CF, Jankaew A, Tsai MC, Immediate effects of thoracic mobilization versus soft tissue release on trunk motion, pain, and lumbar muscle activity in patients with chronic low back pain. J Bodyw Mov Ther 40 (2024): 1664–1671. 39593506 10.1016/j.jbmt.2024.09.004

[50] Sakaguchi T, Gunjotikar S, Tanaka M, Evaluation and Rehabilitation after Adult Lumbar Spine Surgery. Journal of Clinical Medicine 13 (12024): 2915. 10.3390/jcm13102915 PMC11122457 38792457

[51] Slater J, Kolber MJ, Schellhase KC, The Influence of Exercise on Perceived Pain and Disability in Patients With Lumbar Spinal Stenosis: A Systematic Review of Randomized Controlled Trials. Am J Lifestyle Med 10 (2015): 136–147. 30202267 10.1177/1559827615571510 PMC6125093

[52] Kim ER, Kang MH, Kim YG, Effects of a Home Exercise Program on the Self-report Disability Index and Gait Parameters in Patients with Lumbar Spinal Stenosis. J Phys Ther Sci 26 (2014): 305–307. 24648654 10.1589/jpts.26.305 PMC3944311

[53] Hlaing SS, Puntumetakul R, Khine EE, Effects of core stabilization exercise and strengthening exercise on proprioception, balance, muscle thickness and pain related outcomes in patients with subacute nonspecific low back pain: a randomized controlled trial. BMC Musculoskelet Disord 22 (2021): 998. 34847915 10.1186/s12891-021-04858-6 PMC8630919

[54] Sayed D, Grider J, Strand N, The American Society of Pain and Neuroscience (ASPN) Evidence-Based Clinical Guideline of Interventional Treatments for Low Back Pain. J Pain Res. 15 (2022): 3729–3832. Erratum in: J Pain Res 15 (2022): 4075–4076. 36510616 10.2147/JPR.S386879 PMC9739111

[55] Sato Naoto, Sekiguchi Miho, Kikuchi Shinichi, Effects of Long-Term Corset Wearing on Chronic Low Back Pain. Fukushima Journal of Medical Science 58 (2012): 60–65. 22790893 10.5387/fms.58.60

[56] Gignoux Paul, Lanhers Charlotte, Dutheil Frédéric, Non-rigid lumbar supports for the management of non-specific low back pain: A literature review and meta-analysis. Annals of Physical and Rehabilitation Medicine 65 (2022): 101406. 32561503 10.1016/j.rehab.2020.05.010

[57] Calmels P, Queneau P, Hamonet C, Effectiveness of a Lumbar Belt in Subacute Low Back Pain: An Open, Multicentric, and Randomized Clinical Study. Spine 34 (2009): 215–220. 19179915 10.1097/BRS.0b013e31819577dc

[58] Wu L, Munakomi S, Cruz R. Lumbar Spinal Stenosis. [Updated 2024 Jan 30]. In: StatPearls [Internet]. Treasure Island (FL): StatPearls Publishing; (2024) Jan-. Available from: https://www.ncbi.nlm.nih.gov/books/NBK531493/. 30285388

[59] Yoshihara H Indirect decompression in spinal surgery. Journal of Clinical Neuroscience 44 (2017): 63–68. 28688624 10.1016/j.jocn.2017.06.061

[60] Wu AM, Zou F, Cao Y, Lumbar spinal stenosis: an update on the epidemiology, diagnosis and treatment. AME Medical Journal 2 (2017): 63.

[61] Yone K, Sakou T, Kawauchi Y, Indication of fusion for lumbar spinal stenosis in elderly patients and its significance. Spine (Phila Pa 1976) 21 (1996): 242–248. 8720411 10.1097/00007632-199601150-00016

[62] Chen H, Kelling J. Mild procedure for lumbar decompression: a review. Pain Practice 13 (2013): 146–153. 22726247 10.1111/j.1533-2500.2012.00574.x

[63] Binder DK, Schmidt MH, Weinstein PR. Lumbar spinal stenosis. Seminars in Neurology 22 (2002): 157–166. 12524561 10.1055/s-2002-36539

[64] Estefan M, Munakomi S, Camino Willhuber GO. Laminectomy. [Updated 2023 Aug 13]. In: StatPearls [Internet]. Treasure Island (FL): StatPearls Publishing; 2024 Jan-. Available from: https://www.ncbi.nlm.nih.gov/books/NBK542274/. 31194414

[65] Williams MG, Wafai AM, Podmore MD. Functional outcomes of laminectomy and laminotomy for the surgical management of lumbar spine stenosis. Journal of Spine Surgery 3 (2017): 580–586. 29354735 10.21037/jss.2017.10.08 PMC5760430

[66] Fischgrund JS, Mackay M, Herkowitz HN, Degenerative lumbar spondylolisthesis with spinal stenosis: a prospective, randomized study comparing decompressive laminectomy and arthrodesis with and without spinal instrumentation. Spine (Phila Pa 1976) 22 (1997): 2807–2812. 9431616 10.1097/00007632-199712150-00003

[67] Ghogawala Z, Dziura J, Butler WE, Laminectomy plus Fusion versus Laminectomy Alone for Lumbar Spondylolisthesis. New England Journal of Medicine 374 (2016): 1424–1434. 27074067 10.1056/NEJMoa1508788

[68] Baghdadi YM, Moussallem CD, Shuaib MA, Lumbar Spinous Process–Splitting Laminoplasty: A Novel Technique for Minimally Invasive Lumbar Decompression. Orthopedics 39 (2016): e950–e956. 27337665 10.3928/01477447-20160616-03

[69] Kanbara S, Yukawa Y, Ito K, Surgical outcomes of modified lumbar spinous process-splitting laminectomy for lumbar spinal stenosis. Journal of Neurosurgery Spine 22 (2015): 353–357. 25594729 10.3171/2014.9.SPINE1457

[70] Watanabe K, Matsumoto M, Ikegami T, Reduced postoperative wound pain after lumbar spinous process-splitting laminectomy for lumbar canal stenosis: a randomized controlled study. 10.3171/2010.9.SPINE09933 21142464

[71] Rajasekaran S, Thomas A, Kanna RM, Lumbar spinous process splitting decompression provides equivalent outcomes to conventional midline decompression in degenerative lumbar canal stenosis: a prospective, randomized controlled study of 51 patients. Spine (Phila Pa 1976) 38 (2013): 1737–1743. 23797498 10.1097/BRS.0b013e3182a056c1

[72] Hejazi N, Witzmann A, Hergan K, Combined transarticular lateral and medial approach with partial facetectomy for lumbar foraminal stenosis. Technical note. Journal of Neurosurgery 96 (2002): 118–121. 11795699 10.3171/spi.2002.96.1.0118

[73] Kang K, Rodriguez-Olaverri JC, Schwab F, Partial facetectomy for lumbar foraminal stenosis. Advances in Orthopedics (2014): 534658. 25110591 10.1155/2014/534658 PMC4119622

[74] Weinstein JN, Lurie JD, Tosteson TD, Surgical vs nonoperative treatment for lumbar disk herniation: the Spine Patient Outcomes Research Trial (SPORT) observational cohort. JAMA 296 (2006): 2451–2459. 17119141 10.1001/jama.296.20.2451 PMC2562254

[75] Weinstein JN, Tosteson TD, Lurie JD, Surgical vs nonoperative treatment for lumbar disk herniation: the Spine Patient Outcomes Research Trial (SPORT): a randomized trial. JAMA 296 (2006): 2441–2450. 17119140 10.1001/jama.296.20.2441 PMC2553805

[76] Weinstein JN, Tosteson TD, Lurie JD, Surgical versus nonoperative treatment for lumbar spinal stenosis four-year results of the Spine Patient Outcomes Research Trial. Spine (Phila Pa 1976) 35 (2010): 1329–1338. 20453723 10.1097/BRS.0b013e3181e0f04d PMC3392200

[77] Amundsen T, Weber H, Nordal HJ, Lumbar spinal stenosis: conservative or surgical management? A prospective 10-year study. Spine 25 (2000): 1424–1435. 10828926 10.1097/00007632-200006010-00016

[78] Weinstein JN, Tosteson TD, Lurie JD, SPORT Investigators. Surgical versus nonsurgical therapy for lumbar spinal stenosis. New England Journal of Medicine 358 (2008): 794–810. 18287602 10.1056/NEJMoa0707136 PMC2576513

[79] Brown LL. A double-blind, randomized, prospective study of epidural steroid injection vs. the mild procedure in patients with symptomatic lumbar spinal stenosis. Pain Practice 12 (2012): 333–341. 22272730 10.1111/j.1533-2500.2011.00518.x

[80] Zucherman JF, Hsu KY, Hartjen CA, A prospective randomized multi-center study for the treatment of lumbar spinal stenosis with the X STOP interspinous implant: 1-year results. European Spine Journal 13 (2004): 22–31. 14685830 10.1007/s00586-003-0581-4 PMC3468027

[81] Zaina F, Tomkins-Lane C, Carragee E, Surgical versus non-surgical treatment for lumbar spinal stenosis. Cochrane Database Syst Rev 1 (2016): CD010264. 10.1002/14651858.CD010264.pub2 PMC6669253 26824399

[82] Malmivaara A, Slätis P, Heliövaara M, ; Finnish Lumbar Spinal Research Group. Surgical or nonoperative treatment for lumbar spinal stenosis? A randomized controlled trial. Spine (Phila Pa 1976) 32 (2007): 1–8. 17202885 10.1097/01.brs.0000251014.81875.6d

[83] Lee CK, Choi SK, Shin DA, Influence of diabetes mellitus on patients with lumbar spinal stenosis: A nationwide population-based study. PLoS One 14 (2019): e0213858. 30875413 10.1371/journal.pone.0213858 PMC6420006

[84] Deyo RA, Hickam D, Duckart JP, Complications after surgery for lumbar stenosis in a veteran population. Spine (Phila Pa 1976) 38 (2013): 1695–1702. 23778366 10.1097/BRS.0b013e31829f65c1 PMC3865062

[85] Papavero L, Thiel M, Fritzsche E, Lumbar spinal stenosis: prognostic factors for bilateral microsurgical decompression using a unilateral approach. Neurosurgery 65 (2009): 182–187. 19934993 10.1227/01.NEU.0000341906.65696.08

[86] Weiner DK, Holloway K, Levin E, Identifying biopsychosocial factors that impact decompressive laminectomy outcomes in veterans with lumbar spinal stenosis: a prospective cohort study. Pain 162 (2021): 835–845. 32925594 10.1097/j.pain.0000000000002072

[87] Sinikallio S, Aalto T, Airaksinen O, Depression is associated with a poorer outcome of lumbar spinal stenosis surgery: a two-year prospective follow-up study. Spine (Phila Pa 1976) 36 (2011): 677–682. 21037530 10.1097/BRS.0b013e3181dcaf4a

[88] Li G, Patil CG, Lad SP, Effects of age and comorbidities on complication rates and adverse outcomes after lumbar laminectomy in elderly patients. Spine (Phila Pa 1976) 33 (2008): 1250–1255. 18469700 10.1097/BRS.0b013e3181714a44

[89] Raffo CS, Lauerman WC. Predicting morbidity and mortality of lumbar spine arthrodesis in patients in their ninth decade. Spine (Phila Pa 1976) 31 (2006): 99–103. 16395185 10.1097/01.brs.0000192678.25586.e5

[90] Liang H, Lu S, Jiang D, Clinical outcomes of lumbar spinal surgery in patients 80 years or older with lumbar stenosis or spondylolisthesis: a systematic review and meta-analysis. European Spine Journal 29 (2020): 2129–2142. 31912292 10.1007/s00586-019-06261-1

[91] Jensen RK, Jensen TS, Koes B, Prevalence of lumbar spinal stenosis in general and clinical populations: a systematic review and meta-analysis. European Spine Journal 29 (2020): 2143–2163. 32095908 10.1007/s00586-020-06339-1

[92] Kovacs FM, Urrútia G, Alarcón JD. Surgery versus conservative treatment for symptomatic lumbar spinal stenosis: a systematic review of randomized controlled trials. Spine (Phila Pa 1976) 36 (2011): E1335–E1351. 21311394 10.1097/BRS.0b013e31820c97b1

[93] Bays A, Stieger A, Held U, The influence of comorbidities on the treatment outcome in symptomatic lumbar spinal stenosis: A systematic review and meta-analysis. North American Spine Society Journal 6 (2021): 100072. 35141637 10.1016/j.xnsj.2021.100072 PMC8820012

[94] Sudo H, Miyakoshi T, Watanabe Y, Protocol for treating lumbar spinal canal stenosis with a combination of ultrapurified, allogenic bone marrow-derived mesenchymal stem cells and in situ-forming gel: a multicentre, prospective, double-blind randomised controlled trial. BMJ Open 13 (2023): e065476. 10.1136/bmjopen-2022-065476 PMC9896178 36731929

[95] Shabat S, Miller LE, Block JE, Minimally invasive treatment of lumbar spinal stenosis with a novel interspinous spacer. Clinical Interventions in Aging 6 (2011): 227–233. 21966217 10.2147/CIA.S23656 PMC3180519

[96] Coric D, Nassr A, Kim PK, Prospective, randomized controlled multicenter study of posterior lumbar facet arthroplasty for the treatment of spondylolisthesis. Journal of Neurosurgery Spine 38 (2022): 115–125. 36152329 10.3171/2022.7.SPINE22536

[97] Patel VV, Whang PG, Haley TR, Two-year clinical outcomes of a multicenter randomized controlled trial comparing two interspinous spacers for treatment of moderate lumbar spinal stenosis. BMC Musculoskeletal Disorders 15 (2014): 221. 24996648 10.1186/1471-2474-15-221 PMC4109165

